# Immunological analysis of Blau syndrome, a unique autoinflammatory state

**DOI:** 10.3389/fimmu.2026.1793228

**Published:** 2026-04-07

**Authors:** Atika Dhar, Atsushi Kitani, Warren Strober

**Affiliations:** Mucosal Immunity Section, Laboratory of Clinical Immunology and Microbiology, National Institute of Allergy and Infectious Diseases (NIAID), National Institutes of Health (NIH), Bethesda, MD, United States

**Keywords:** Blau syndrome, Card15, NOD2, auto-inflammation, inflammatory disease

## Abstract

Autoinflammation typically arises from mutations affecting molecules such as inflammasome backbones that give rise to gain-of-function (GOF) pro-inflammatory activity requiring little or no normal ligand stimulation. This has been assumed to be the case in the auto-inflammation known as Blau syndrome wherein mutations usually present in the nucleotide oligomerization domain of the *CARD15* gene encoding NOD2 result in widespread granulomatous inflammation, seemingly in the absence of NOD2 stimulation by its canonical ligand, muramyl dipeptide (MDP); moreover, despite such lack of ligand stimulation, NOD2 bearing a Blau mutation is thought to cause inflammation by initiating conventional downstream signaling that ultimately results in NF-κB activation. However, newer data concerning Blau syndrome pathogenesis suggest a more complex picture in which Blau *CARD15* mutations cause inflammation by unconventional and/or loss of conventional signaling and which depend, at least in part, from a genetic defect which arises from loss-of-function pro-inflammatory activity. In this review, we present and analyze these newer data with the aim of defining a further pathway to the understanding and treatment of this disease.

## Introduction

Blau syndrome is a rare autoinflammatory disease caused by autosomal dominant mutations occurring in the nuclear oligomerization (NOD) domain of the *CARD15* gene, the gene encoding the peptidoglycan sensor NOD2. This disease is therefore genetically related to Crohn’s disease which is also sometimes associated with *CARD15* genetic abnormalities, in this case polymorphisms mainly located downstream of the NOD domain in the *CARD15* leucine-rich repeat (LRR) domain. Of interest, it has recently become apparent that Blau syndrome is also related to another autoinflammatory disease known as Yao syndrome which is again caused by *CARD15* mutations in the *CARD15* NOD domain; however, in this case the mutations are present at intron and exon sites generally downstream of Blau mutation sites. Despite this genetic similarity, Blau syndrome and Yao syndrome are quite distinct clinical entities, and both do not display the gut-centered features of Crohn’s disease. This may relate to the fact that normal NOD2 function is multifaceted and thus genetically determined NOD2 abnormalities have the potential to cause alterations centering on different facets of such function.

Blau syndrome was first described simultaneously by two separate groups, one led by Edward Blau and the other by Douglas Jabs (1, 2). These reports defined Blau syndrome as an early onset familial disease and noted that its main pathological manifestation, widespread granulomatous inflammation, typically gives rise to the clinical triad of arthritis, skin rash and uveitis. However, in later studies, it became apparent that NOD2 Blau mutations could lead to granulomatous inflammation in virtually any area of the body in particular subsets of patients (3–5).

Uveitis proved to be the most consequential of these manifestations inasmuch as it can lead to a level of visual impairment severely impacting quality of life; as a result, it is a major determinant of treatment success (6, 7). Whereas cases of Blau syndrome are not infrequently reported, it has a low incidence rate of 0.06 per 100,000 person-years (8). This includes cases with familial disease as well as those with sporadic disease initially called early onset sarcoidosis because Blau syndrome and sarcoidosis are both characterized by granulomatous inflammations; however, it is now recognized that Blau syndrome and sarcoidosis are pathologically distinct and differ both in organ involvement and disease course as well (9).

Mutations underlying Blau syndrome are commonly thought to cause a gain-of-function (GOF) immunologic abnormality similar to those occurring as a result of mutations of genes affecting inflammasome function. This is in contrast to the mutations associated with Crohn’s disease which are thought to cause a loss-of-function (LOF) immunologic abnormality. This characterization of Blau or Crohn’s *CARD15* mutations fits with the fact that, as discussed below, the location of Blau mutations in the *CARD15* gene encoding NOD2 results in NOD2 with potential of spontaneous activation whereas the location of the Crohn’s disease mutations results in NOD2 unable to respond to ligand and inactivation. However, these GOF/LOF characterizations are perhaps misleading when applied to regulatory immune function defects in the two diseases because loss or gain of regulation can ultimately result in LOF-associated increases or GOF-associated decreases in final inflammatory responses. In the review of Blau syndrome presented below, we will examine all major aspects of the immunologic dysfunction so far reported in this disease with aim of understanding the immunologic factors driving the inflammation in this disease. This will enable us to re-examine the Blau syndrome gain-of-function hypothesis with respect to whether it solely or partially describes the primary defect in Blau syndrome.

## Genetic abnormalities associated with Blau syndrome

The genetic abnormality underlying Blau syndrome was first reported by Miceli-Richard et al., who sequenced *CARD15* exons in three Blau families because this gene mapped to a susceptibility locus at chromosome location 16q12.1–13 previously identified in a large Blau kindred by linkage analysis (10). They identified three different autosomal dominant missense *CARD15* mutations, two of which (R334Q and R334W) proved subsequently to be the most common mutations associated with Blau syndrome, occurring in about 40-80% of patients. These *CARD15* mutations were clearly distinct from the single nucleotide polymorphisms (SNPs) associated with Crohn’s disease since they were present in the nucleotide binding domain (NBD) of *CARD15* rather than in the down-stream leucine-rich repeat or LRR domain. In addition, screening of a large group of Crohn’s patients did not identify patients with Blau-like mutations. Whereas the gene mutations identified in this study of patients with Blau syndrome were clearly inherited abnormalities, in later studies patients with sporadic gene mutations were also identified; the latter are commonly referred to as patients with “early onset sarcoidosis” (EOS). Whereas this designation is justified by the fact that both Blau syndrome and sarcoidosis are characterized by non-caseating granulomas, these diseases are genetically distinct and pathologically/clinically distinct as well (9).

**Figure 1:**
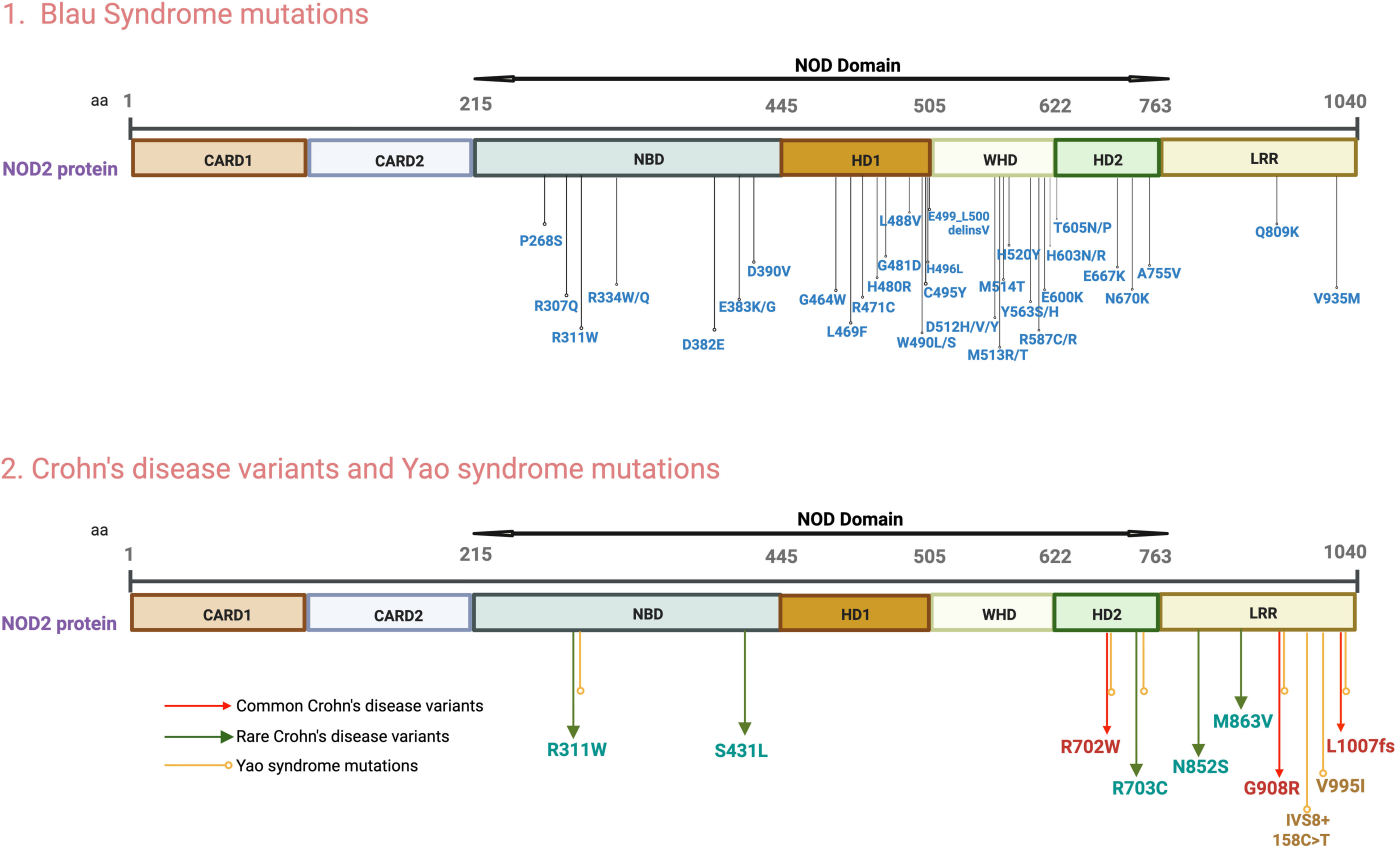
*CARD15* Blau syndrome mutations, Yao syndrome mutations and Crohn’s disease variants. This image depicts the various domains of the *CARD15* gene and the location of mutations within these domains that cause abnormalities in NOD2 protein function. Upper panel: Blau syndrome mutations; Lower panel: Yao syndrome mutations or common and rare Crohn’s disease variants. Image was generated using Biorender software.

Since this initial study between 40–50 additional autosomal dominant gene mutations have been identified in Blau syndrome patients. The majority are located within the nucleotide binding and oligomerization domain (NOD) of *CARD15*, a multiplex domain comprised by the aforementioned nucleotide binding domain (NBD) as well as the helical domain 1 (HD1), the winged helical domain (WHD) and the helical domain 2 (HD2). However, at least two mutations (Q809K and V935M) are located well outside the NOD domain, within the downstream LRR domain (**Figure 1**). These latter (LRR) mutations occurred in patients with Blau syndrome-like uveitis and/or dermatitis/arthritis and in the absence of GI symptoms suggestive of Crohn’s disease (11, 12). In the case of the V935M mutation, the mutation was also extensively characterized as pathologic by several algorithms and tracked with disease occurrence in siblings. The significance of these “atypical” non-NOD domain mutations lies in the fact that Blau mutations do not necessarily cause disease by a direct effect on ADP binding ordinarily occurring in the NOD region that impairs the latter’s regulation of NOD2 unfolding and oligomerization, prior to RIPK2 binding (see Discussion below). On the contrary, mutations can presumably have this effect more indirectly by simply causing conformational changes. All of the Blau *CARD15* defects were missense mutations except one which consisted of six base c.1493_1498 base deletion resulting in a Val insertion in place of Glu-Leu at the mutated (499-500) site (**Figure 1**). The patient with this mutation exhibited severe uveitis along with other typical Blau symptoms and was said to have EOS (13).

NOD2 mutations in the NOD domain underlying Blau syndrome (14–17) (**Figure 1**) are true mutations in the sense that they are abnormalities that rarely occur in the general population and when they do occur, they are highly penetrant and usually cause disease. This is in contrast to NOD2 polymorphisms in the LRR domain underlying some cases of Crohn’s disease since the latter are abnormalities that do occur in the general population with some frequency and in most cases are not associated with the occurrence of disease; evidently, Blau mutations are sufficient in themselves to cause at least some manifestations of Blau syndrome whereas Crohn’s polymorphisms require the presence of cooperating genetic abnormalities. This said, it should be pointed out that in several families a NOD2 mutation causing typical Blau syndrome symptoms in one or more members of a kindred did not do so in others; these mutations were not atypical in location and in fact occurred in one family with the common R334W mutation; thus a Blau mutation was not necessarily penetrant in all cases and may sometimes require the presence of other genetic abnormalities, as in the case of the Crohn’s disease polymorphisms (18–20).

The NOD2 mutations underlying Blau syndrome have been correctly considered dominant mutations inasmuch as they are capable of causing inflammatory disease in heterozygous carriers with normal NOD2 function potentially exerted by the unmutated NOD2 gene on the unaffected allele. It is important to add, however, that the NOD2 mutation is not only dominant it is dominant negative in that the mutated gene exerts an inhibitory effect on the function of the normal NOD2 gene. This is supported by the fact that macrophages or dendritic cells from Blau syndrome patients or mice bearing a Knock-In (KI) Blau mutation on one allele consistently exhibit reduced cytokine responses following stimulation by MDP (NOD2 ligand) that are much lower than the 50% reduction predicted by loss of one allele ( (21); Dhar et al., 2025, unpublished). It is also supported by the fact that the Blau-mutated gene product (mutated NOD2) interferes with and/or negates the “cross-regulatory” function of NOD2 signaling whereby the latter moderates TLR ligand-induced responses and their pro-inflammatory sequelae. On this basis, mice bearing a transgene expressing a Blau mutation are not protected from the development of induced colitis (TNBS-colitis) as are mice bearing a wild type (WT) transgene; similarly, mice bearing a KI Blau mutation on one chromosome are less protected from induced colitis (DSS-colitis) upon enhanced NOD2 stimulation by intraperitoneal administration of MDP (21). Thus, in both cases the presence of NOD2 produced by the gene with a Blau mutation interferes with the cross-regulatory function of the NOD2 produced by the unmutated NOD2 allele and is exhibiting a dominant negative effect.

Relating to Blau mutation penetrance is the question of whether environmental factors contribute to the onset and/or persistence of disease in Blau syndrome patients and therefore determine whether existing Blau mutations are expressed. In most cases of Blau syndrome disease onset occurs early in life and is not associated with obvious infection (i.e., onset of disease symptoms appears to be spontaneous); thus, it seems likely that in most cases ubiquitous, ordinarily non-pathogenic environmental exposures are sufficient to initiate disease. The possible exceptions to this rule are the several cases in which disease onset has been noted to follow BCG vaccination (22, 23); or in the one case in which *Propionibacterium acnes* was identified in granulomatous skin lesions of the patient (24). Since the organisms involved in these cases express MDP, the major NOD2 ligand, it was suggested that these organisms cause disease by MDP activation of the mutant Blau NOD2 but this is questionable because NOD2 with a Blau mutation is poorly responsive to MDP. Late- or adult-onset Blau syndrome patients are theoretically more likely to have had an environmental precipitant. Nevertheless, in the one report of late onset Blau syndrome in which no symptoms attributable to Blau syndrome were found prior to onset of BS disease (uveitis) no precipitating factors were in fact observed (25).

The main *CARD15* polymorphisms/mutations initially shown to increase risk for occurrence of Crohn’s disease (risk variants), as alluded to above, differ from those in Blau syndrome in that they are located in or near the LRR domain. These polymorphisms (R702W, G908R and L1007fs) differ in location from that of the two reported Blau syndrome LRR mutations (Q809K and V935M). More recently, deep resequencing studies have revealed the presence of rare risk variants not associated with the original triad of polymorphisms and located in the NOD domain as well as in the LRR domain (R311W, S431L, R703C, N852S, M863V) (26) (**Figure 1**). In addition, numerous other rare risk variants occurring in all parts of the NOD2 molecule have been identified that may cause disease (in both early onset CD patients or adult CD patients) independently of the main risk polymorphisms when inherited in trans as recessive homozygous or compound heterozygous variants (27). Alternatively, it is possible (but not proven) that some of these or other widely distributed rare variants contribute to disease as amplifiers of the effects of the major risk polymorphisms even when present in cis (28). In any case, these rare and atypical CD risk variants are distinct from Blau mutations even when present in the NOD domain. It is therefore apparent that whereas there is little locational overlap between Blau mutations and major (common) Crohn’s disease polymorphisms there is such overlap between Blau mutations and rare Crohn’s disease polymorphisms; thus, the functional effect of a NOD2 mutation/polymorphism is independent of location, at least to some extent. This is highlighted by the report of one case of early onset Crohn’s disease with a homozygous recessive mutation in the NOD domain (R426H) not found in Blau syndrome and causing increased NOD2 responsiveness to MDP (29).

In recent years a new NOD2 autoinflammatory disease has been identified and extensively described by Yao and his co-workers. This syndrome, appropriately called Yao syndrome, is clinically and pathologically distinct from Blau syndrome and is caused by mutations somewhat downstream of those causing Blau syndrome (30). Patients with this syndrome exhibit a wide spectrum of symptoms including recurrent fever/night sweats, arthralgias without joint deformity, eyelid swelling, distal leg swelling, GI symptoms including pain and diarrhea but without Crohn’s-like infiltrates, chest pain and pericarditis and drug or other allergies. The granulomatous inflammation affecting eyes, skin and joints common to Blau syndrome patients is not present. More than half of Yao patients express an intronic mutation (alone or in association with another mutation) in the intronic segment following the 8^th^ *CARD15* exon (IVS8 + 158C>T) that has no known effect on NOD2 expression or RNA transcript splicing (31). Other mutations occurring alone or with IVS8 + 158C>T include R702W, L1007fs, G908R, R311W, R703C and V995I; finally, one patient with a R311W and R703C dual mutation was also observed (30, 32) (**Figure 1**). Thus, Yao syndrome mutations are generally distinct from Blau syndrome mutations in that they are mainly present in a more downstream part of the NOD domain or as a mutation in a non-coding or coding region of LRR itself and are in each case not duplicative of Blau mutations. Remarkably, several Yao patients bear the distinctive L1007fs mutation associated with Crohn’s disease yet display a disease unlike Crohn’s disease. This observation is a clear indication that the Crohn’s disease risk polymorphisms require the presence of additional genetic abnormalities and/or environmental factors to cause disease and are “plastic” with regard to the type of disease they induce.

The fact that Blau syndrome-associated mutations, while distinctive, are sometimes locationally interspersed among Yao mutations on the one hand and Crohn’s polymorphisms on the other, suggests that *CARD15* autoinflammation is caused by subtle abnormalities of *CARD15* structure that are not easily predicted by location of mutation. This conclusion applies to the variations in the clinical manifestations defining *CARD15* autoinflammation disease categories as well as to variation in the clinical manifestations occurring within disease categories. With regard to the latter, attempts to predict onset, severity or location of disease in Blau syndrome patients based on mutational location has been largely unrewarding. Thus, while it was reported that the common R334W Blau mutation occurring in a Japanese Blau cohort was marked by increased severity and frequency of ocular manifestations (33), this correlation was not observed in the large, mainly European, Blau cohort (34). This ethnic discrepancy hints that additional and associated genetic abnormalities already alluded to above help shape how mutations are expressed in individual patients. Finally, it should be mentioned that gene dosage operating through genetic mosaicism may affect disease expression in some Blau kindreds. This possibility has already been reported to occur in a family consisting of a father who expressed a Blau mutation in a relatively low percentage of hematopoietic cells and two of his children who expressed the same mutation in the expected half of their hematopoietic cells (35). The father, presumed to have a gonadal somatic (“gonosomic”) Blau mutation, had late onset, mild disease limited to eye and skin inflammation, whereas the children, presumed to have a mutation inherited from the father, had full-fledged early onset and severe Blau syndrome disease characterized by the classic Blau triad of symptomatology. Thus, in this family the onset and severity of disease was determined by mosaicism and gene dosage.

The most important question regarding Blau mutations in *CARD15* is how and why these mutations result in granulomatous autoinflammation affecting multiple tissues. This question is best broken into two parts: first, how does the mutation affect the canonical NOD2 signaling pathway and, second, how does it cause pro-inflammatory (aberrant) signaling possibly independent of the canonical NOD2 signaling pathway? We will consider the first part here and the second part below as part of a discussion of the immunologic mechanisms underlying Blau syndrome.

The generally accepted model of NOD2 activation, derived from earlier studies of NLRC4 (Apaf-1) activation, holds that inactive NOD2 exists in a folded configuration that shields the NOD2 CARD domains from binding to itself or to RIPK2 via CARD/CARD interactions, thus preventing an interaction that would initiate RIPK2 oligomerization and downstream signaling (36, 37). From this it follows that ligand-induced NOD2 activation involves disassembly of the folded configuration and freeing NOD2 CARD domains for self-interaction or interaction with RIPK2 CARD domains. ADP plays a key role in the molecular mechanism underlying this activation model because when embedded in the NOD domain of NOD2 it acts as a draw-string that maintains NOD2 in a closed configuration. As an extension of this concept, ligand activation of NOD2 involves exchanging ADP with ATP, the latter having no draw-string effect and allowing NOD2 unfolding.

Recent crystallographic studies have led to the identification of the several points of interaction and hydrogen bond formation between ADP and amino-acids in the NBD, HD1 and WHD subdomains of the NOD2 NOD domain that enable ADP drawstring function and NOD2 folding (38). In addition, these studies have allowed identification of NOD subdomain interactions that occur secondary to the ADP draw-string effect that further support NOD2 folding, including those between the NBD and HD2 subdomains. These findings led directly to the observation that the amino-acids participating in the maintenance of the ADP-enforced folded configuration were frequently those subject to mutational substitution by Blau mutations. This, in turn, led to the strong presumption that the mutations, each in its own way, undermine the drawstring function of ADP and result in a NOD2 in a perpetually open configuration subject to spontaneous activation.

In other studies providing further support for the above concept of Blau mutational effects it was found that some Blau mutations occur in the “Walker B Box” region of the NOD subdomain, a conserved region shown to have ATP binding and hydrolysis properties (39, 40). Such mutations were postulated to cause NOD2 to assume a perpetually open configuration following ligand interaction because ATP associated with the activated molecule cannot be hydrolyzed and converted back to ADP having draw-string function. This explanation of mutational dysfunction in Blau syndrome applies only to mutations in the NOD domain affecting ATP hydrolysis and thus cannot explain the effect of Blau mutations not in the region having such ATPase hydrolysis function as does the mutational effect of ADP draw-string function discussed previously. In addition, it differs from the ADP draw-string dysfunction explanation by the fact that in this case the mutation allows NOD2 activation of as yet non-activated NOD2 that has some ability to respond to MDP stimulation, whereas draw-string dysfunction posits complete MDP unresponsiveness due to already activated NOD2.

These complementary and seemingly definitive molecular explanations of Blau syndrome pathogenesis, however, do not satisfy key facts concerning the function of NOD2 bearing Blau syndrome mutations (21). Most importantly, it has been shown that plasmids expressing common Blau mutations yield NOD2 proteins that exhibit reduced binding to one another or to unmutated NOD2. In addition, similar plasmids yield NOD2 protein that does not bind to RIPK2 or binds but does not result in a RIPK2 capable of ubiquitination (21). Finally, and most importantly, in several studies, PBMCs, macrophages or dendritic cells from Blau patients generally do not exhibit high baseline cytokine levels and respond poorly to NOD2 ligand stimulation (21, 41–46). These characteristics of Blau syndrome NOD2 predict that Blau mutations cause changes that result in a form of NOD2 largely incapable of normal signaling activities initiated by MDP; at the same time, they predict that Blau mutations result in a NOD2 incapable of exerting regulatory functions that rein in excessive and pro-inflammatory myeloid and T cell responses. Thus, these characteristics throw into question the GOF paradigm of Blau mutations asserting that the latter simply result in a perpetually activated NOD2 with primary pro-inflammatory activity. In the Sections that follow, we turn our attention to these issues with a full discussion of studies describing the immunological responses in Blau syndrome.

### Blau syndrome and NF-κB activation

Blau syndrome is generally considered an autoinflammatory disease in the sense that it is thought to be caused by the unfettered activity of an innate signaling molecule, in this case NOD2, that occurs independently of stimulation by its usual ligand in this case, a peptide derived from a bacterial peptidoglycan, MDP. Importantly, in this formulation of Blau syndrome autoinflammation the latter is similar to NLRP3 inflammasome-mediated autoinflammation in that it occurs via pro-inflammatory signaling that is not essentially different from normal pro-inflammatory signaling and differs from normal signaling only in magnitude and/or persistence. Two inter-locking strands of research support this view.

First, there are structural studies, already reviewed above, showing that *CARD15* mutations result, in one way or another, in a NOD2 that has assumed an activated (unfolded) posture ordinarily occurring only after NOD2 interaction with MDP (or another unknown ligand) and that is therefore capable of down-stream signaling in the absence of ligand. Second, we have functional studies of NOD2 protein bearing Blau mutations (Blau NOD2) that suggest that Blau NOD2 is capable of inducing ligand-independent NF-κB activation. These studies, conducted repeatedly and in various laboratories (23, 39, 40, 47–50), consist mainly of studies showing that Blau NOD2 causes NF-κB activation when over-expressed by a Blau NOD2 plasmid transfected into a cell line (HEK293 cells) and cultured in the absence of MDP; in contrast, Wild Type (WT, control) or NOD2 bearing an irrelevant mutation not associated with human disease (also expressed by transfected plasmids) (23) do not cause NF-κB activation when expressed and cultured in the same way. Addition of MDP to the cell cultures of cells expressing Blau protein typically causes NF-κB activation 2–3 fold greater than baseline (non-MDP associated and spontaneous) activation but this level of activation is not different from that obtained in cells expressing WT protein (40, 49). Thus, this over-expression system seems to fulfill the expectations of the structural studies in that they show that Blau NOD2 spontaneously activate NF-κB in the absence of MDP stimulation. The fact that MDP does increase NF-κB activation in this system is explainable by assuming that the open configuration is not complete and, thus, that a substantial fraction of the NOD2 is in a normal configuration in spite of the mutation and is still subject to MDP-mediated activation.

The above molecular and functional explanation of Blau syndrome autoinflammation, while at least superficially complete, remains open to critique and in need of further validation. Thus, whereas the structural data concerning Blau NOD2 configuration leading to the conclusion that Blau NOD2 is potentially capable of MDP-independent activity is well-supported, proof that this potentiality is fulfilled in Blau syndrome patient cells rests mainly in the less definitive data obtained in the studies of Blau NOD2 over-expression in HEK293 cells. The reason to consider the latter studies a “weak link” in the explanation of Blau NOD2 autoinflammation is that if indeed Blau NOD2 exists in an open configuration subject to ligand-free activation, its over-expression in a cell line renders it prone to non-canonical and/or quantitatively non-physiological canonical down-stream signaling and NF-κB activation.

In any case, such signaling would not replicate the actual Blau NOD2 signaling in unstimulated patient cells that contain far lower amounts of NOD2 and can activate negative cellular mechanisms that limit certain pathways of NOD2 activation. Support for this alternative interpretation of the over-expression studies comes from the fact that upon MDP stimulation the Blau-plasmid transfected HEK293 cells exhibited NF-κB activation equivalent to that of WT-plasmid transfected HEK293 cells (40, 49); this is contrary to the far lower cytokine production ordinarily displayed by Blau cells (which should theoretically reflect both spontaneous and induced responses) and thus suggests that the spontaneous NF-κB activation is non-existent in untransfected patient cells.

Further support for this alternative interpretation of the over-expression studies comes from the fact that increased ligand-independent NF-κB activation was not seen in studies of all patients (21, 41–46, 51); in addition (and more importantly) in one study of Blau NOD2 function in Blau NOD2 plasmid-transfected HEK293 cells, in which steps were taken to more closely model physiological conditions such as transfection of low amounts of plasmid, it was found that Blau protein bearing each of six different Blau mutations including the common R334Q and R334W mutations exhibited reduced interaction with RIPK2 in immunoprecipitation studies (21). Furthermore, addition of MDP to HEK293 cultures induced RIPK2 phosphorylation and polyubiquitination of RIPK2 in cells transfected with WT NOD2 plasmids but such phosphorylation and/or ubiquitination was not seen in cells transfected with Blau plasmids. Finally and most tellingly, HEK293 cells transfected with Blau plasmids exhibited baseline (MDP-independent) NF-κB activation equivalent to cells transfected with WT plasmids and did not exhibit NF-κB activation upon MDP stimulation as did cells transfected with WT plasmids (21).

If the HEK293 over-expression system commonly used to support the idea that Blau NOD2 exerts ligand-independent and spontaneous NF-κB activation is suspect as implied by the above considerations, what methodology can be applied in its stead to support this idea more unequivocably? One immediately available answer to this question would be the demonstration that unstimulated, hematopoietic cells obtained directly from patients exhibit evidence of unstimulated NF-κB activation and/or cytokine secretion. In the somewhat sparse group of studies of this kind it was found that macrophages and/or dendritic cells derived from circulating monocytes of five Blau syndrome patients with a variety of NOD2 Blau mutations did not display increased baseline (spontaneous) NF-κB activation nor accompanying unstimulated increased pro-inflammatory cytokine secretion (45, 46, 51). These negative data were buttressed by the finding that neither spontaneous NF-κB activation nor cytokine secretion was exhibited by spleen cells obtained from mice with a Knock-In Blau mutation or in iPSC cells bearing a Blau NOD2 mutation (45, 51).

On the other hand, contrary data was obtained in the study of one patient with Blau syndrome associated with a rare NOD2 mutation (C483W) who was found to have increased percentage of circulating CD11b^+^ cells with positive staining for phosphorylated p65 indicative of NF-κB activation upon flow cytometric analysis as well as accompanying increases in circulating pro-inflammatory cytokines (19). In addition, in another study, two Blau syndrome patients were found with mononuclear circulating cells positive for phosphorylated p65 (Rel A) and p38 as determined in a reverse phase protein array assay; however, the significance of this finding with respect to spontaneous NF-κB activation is clouded by the fact that this was unaccompanied by an expected increase in circulating mononuclear cell levels of major pro-inflammatory cytokines as determined by the same assay (43).

The above studies in which spontaneous NF-κB activation in Blau syndrome patients was measured directly are accompanied by studies of a larger group of patients in which this parameter was measured indirectly by assay of pro-inflammatory cytokine production by unstimulated whole peripheral blood mononuclear cells or purified macrophages and dendritic cells. None of the 21 Blau syndrome patients in this group displayed increased unstimulated cytokine production greater than cells from controls and all displayed lower than control cytokine responses to MDP stimulation and normal or increased cytokine responses to stimulation by a variety of TLR ligands (21, 41–46). In summary, then, the preponderance of available evidence does not support the prevailing conclusion drawn from studies of Blau NOD2 in HEK293 cells that mutated Blau NOD2 induces spontaneous unstimulated NF-κB activation. In view of these data, it seems likely that the over-expression studies result reflects a potential aspect of Blau NOD2 that does not obtain in actual patient cells. One must therefore seek a mechanism other than spontaneous activation of canonical NOD2 signaling to account for the auto-inflammation defining Blau syndrome.

### Blau syndrome and inflammasome-dependent IL-1β production

In hallmark studies of NOD2 responses following its activation by MDP in humans conducted by Hedl and Abraham evidence was obtained that such stimulation caused early secretion of IL-1β and that the latter led to MAP-kinase (p38 and JNK) stimulation and subsequent production of a host of other pro-inflammatory cytokines (52). This early IL-1β secretion was shown to be dependent on caspase-1, suggesting that NOD2 either functions as a typical inflammasome platform itself or interacts with and causes assembly and activation of a known (more *bona fide*) inflammasome platform such as NLRP1 or NLRP3 to produce IL-1β. Indeed, in subsequent studies addressing this possibility, evidence was obtained that NOD2 does in fact interact with and activate the NLRP1 inflammasome (53) as well as, more questionably, the NLRP3 inflammasome (54–57). This body of mostly *in vitro* evidence that IL-1β is a major outcome of normal NOD2 signaling is, as yet, poorly supported by *in vivo* studies of MDP-stimulated inflammatory responses (58); nevertheless, it poses the question of whether Blau NOD2, in the absence of MDP stimulation, causes inflammation by causing excessive IL-1β secretion via intrinsic (self) or extrinsic (interactive) inflammasome activity.

Theoretical evidence that Blau NOD2 was acting as an inflammasome platform did in fact come from the observation that the most common *CARD15* mutations occurring in Blau syndrome (R334W and R334Q) were homologous in molecular domain location to the R260W mutation of NLRP3 causing NLRP3 inflammasome-mediated auto-inflammation (50, 59, 60). However, several studies in which actual IL-1β secretion in Blau syndrome was measured do not support this possibility. Thus, in an early study of a Spanish cohort of Blau syndrome patients, the plasma levels of several cytokines were assessed in two patients carrying the R587C NOD2 mutation. One of the patients, who displayed more severe symptoms, showed increases in plasma levels of IL-6, TNF-α and only a slight increase in IL-1β (61). In further studies, in this case of 5 patients bearing R334W or R334Q NOD2 mutations, the response of Blau syndrome patient peripheral blood mononuclear cells (PBMCs) to MDP and TLR agonists, namely LPS and Pam3Csk4 was assessed. In the absence of any external stimulation, the PBMCs from Blau patients did not secrete enhanced levels of IL-1β, IL-12p40 and TNF-α and the secretion of these cytokines in response to LPS and Pam3Csk4 by Blau patients PBMCs was comparable (TNF-α and IL-1β), or slightly lower (IL-12p40), compared to the controls.

Additionally, in response to MDP, while control PBMCs secreted low but detectable amounts of IL-1β, the IL-1β levels in Blau patient PBMCs were comparable with or without MDP stimulation (41). These findings were essentially duplicated in another study of two Blau syndrome patients bearing R334W mutations in which it was noted that Blau patient PBMCs secreted low amounts of IL-1β in response to MDP and LPS. Moreover, the patient PBMCs did not show any spontaneously increased secretion of IL-1β as compared to the control cells (42). Finally, in yet another study, in this case of three Blau syndrome patients harboring the E383K mutation, the serum cytokine levels of IL-1β, IL-6, IL-8, TNF-α and IFN-γ were comparable between healthy control and Blau syndrome patients; moreover, patient-derived PBMCs did not exhibit basal increases in cytokine secretion and the response to MDP was again comparable to control values for all the cytokines tested except IL-6 which was lower (43).

The above mainly *ex vivo* assessment of IL-1β secretion in Blau syndrome would be strengthened by an *in vivo* assessment, i.e., an evaluation of whether IL-1β is an essential component of Blau inflammation occurring in patients. However, studies of this type consisting of whether or not an available biologic agent blocking IL-1β signaling (anakinra/canakinumab) is an effective treatment of Blau inflammation provide mixed results: in some studies an improvement was observed (61–63) whereas in other studies no improvement was observed (41). These *in vivo* studies are therefore presently inconclusive and, in any case, need to be augmented by more controlled and longer-term studies. Overall, the presently available data rule against IL-1β as a major mechanism of Blau syndrome inflammation, but additional, more targeted studies are necessary to more definitively answer this question.

### Blau syndrome and IFN-γ/TNF-α stimulation

IFN-γ figures to be a cytokine in normal NOD2-mediated myeloid cell function both as a downstream effector and as an upstream regulatory molecule; thus, it may be involved in Blau syndrome (abnormal) NOD2-mediated NOD2 function as well. Its role in normal (MDP-induced) NOD2 effector function was nicely shown in studies of MDP-induced uveitis wherein it was found that the latter was greatly reduced in mice with NOD2 deletion (64). On the other hand, its role as an modulator of normal NOD2 function was demonstrated by its ability to up-regulate NOD2 expression, particularly in synergy with TNF-α and, further, to shift NOD2 cellular distribution into a more pro-active mode (65–67). The corresponding role of IFN-γ as an effector in Blau syndrome seems to be limited inasmuch as in the few studies of IFN-γ levels in patient plasma or IFN-γ production by patient PBMCs no enhancement of IFN-γ production was noted (43, 46). However, its role as an up-stream regulator of Blau syndrome myeloid cell function does appear to be enhanced, as particularly illustrated in studies of iPSC-derived macrophage cell lines (51) to which we will now turn our attention.

In a range of studies conducted by Takeda et al., utilizing control induced pluripotential stem cell-derived macrophages (iPS macrophages) and control iPS macrophages with a R334W Blau mutation or alternatively iPS macrophages from a patient with a R334W mutation and iPS macrophages from the same patient in which the mutation had been corrected to investigate responses to IFN-γ stimulation. None of these cell lines exhibited NF-κB activation (detected by a luciferase reporter) or translocation to the nucleus when cultured without stimulation, confirming results mentioned above showing that the mutation does not cause spontaneous activation. Addition of IFN-γ to cultures (in the absence of MDP) caused definitely enhanced NF-κB activation in control macrophages with a Blau mutation vs control macrophages without a mutation but only weak (albeit significant) enhanced NF-κB activation in iPS macrophages from a Blau patient vs iPS cells in which the mutation had been corrected. Thus, the IFN-γ stimulatory effect on NF-κB activation was tenuous since it was only robust in macrophages with an induced mutation.

This conclusion is strengthened by the fact that in separate studies of macrophages from a Blau patient, culture with IFN-γ caused only a marginal (albeit significant) enhancement of NF-κB activation. Companion studies of the effect of IFN-γ stimulation in the absence of MDP on the various cell line cytokine responses showed that control iPS macrophages bearing a Blau mutation did produce increased amounts of pro-inflammatory cytokines compared to macrophages without this mutation; however, this increased cytokine production was equal to that seen in the comparison of iPS macrophages from a patient with Blau syndrome in which the mutation had been corrected. Thus, in this case the two comparisons were equivalent despite differences in their NF-κB responses. This suggests that the pro-inflammatory outcome of the Blau mutation is heightened by exposure to IFN-γ but, for the most part, this IFN-γ effect is not NF-κB-mediated. This conclusion is consonant with RNAseq studies conducted by these authors showing that IFN-γ stimulation augmented an IFN-γ gene response that was in fact present prior to any stimulation (51).

Further insight into the role of IFN-γ signaling in Blau syndrome came from studies by Kitagawa et al., that focused on TNF-α effects on Blau cell responses. In initial studies these authors compared the response of CD14^+^ monocyte-derived macrophages from a group of Blau syndrome patients who had or had not received anti-TNF-α treatment and found that macrophages from untreated patients exhibited a higher responsiveness to IFN-γ as assessed by the production of IL-6 and TNF-α. These results were in line with RNAseq studies which showed that the two groups of Blau patients differed with respect to transcriptomic landscape in that the anti-TNF-α treated Blau patients resembled that of healthy controls. Additionally, in the non-anti-TNF-α treated Blau patient macrophages baseline *CXCL8, IL1β*, and *CXCL2* levels were increased and they were found to be in an activated state displaying the increased expression of genes related to NF-κB signaling, NLR signaling and PI3K-AKT signaling and the expression of genes downstream of NF-κB signaling such as *TNFAIP3*, in comparison to macrophages from healthy counterparts.

**Figure 2:**
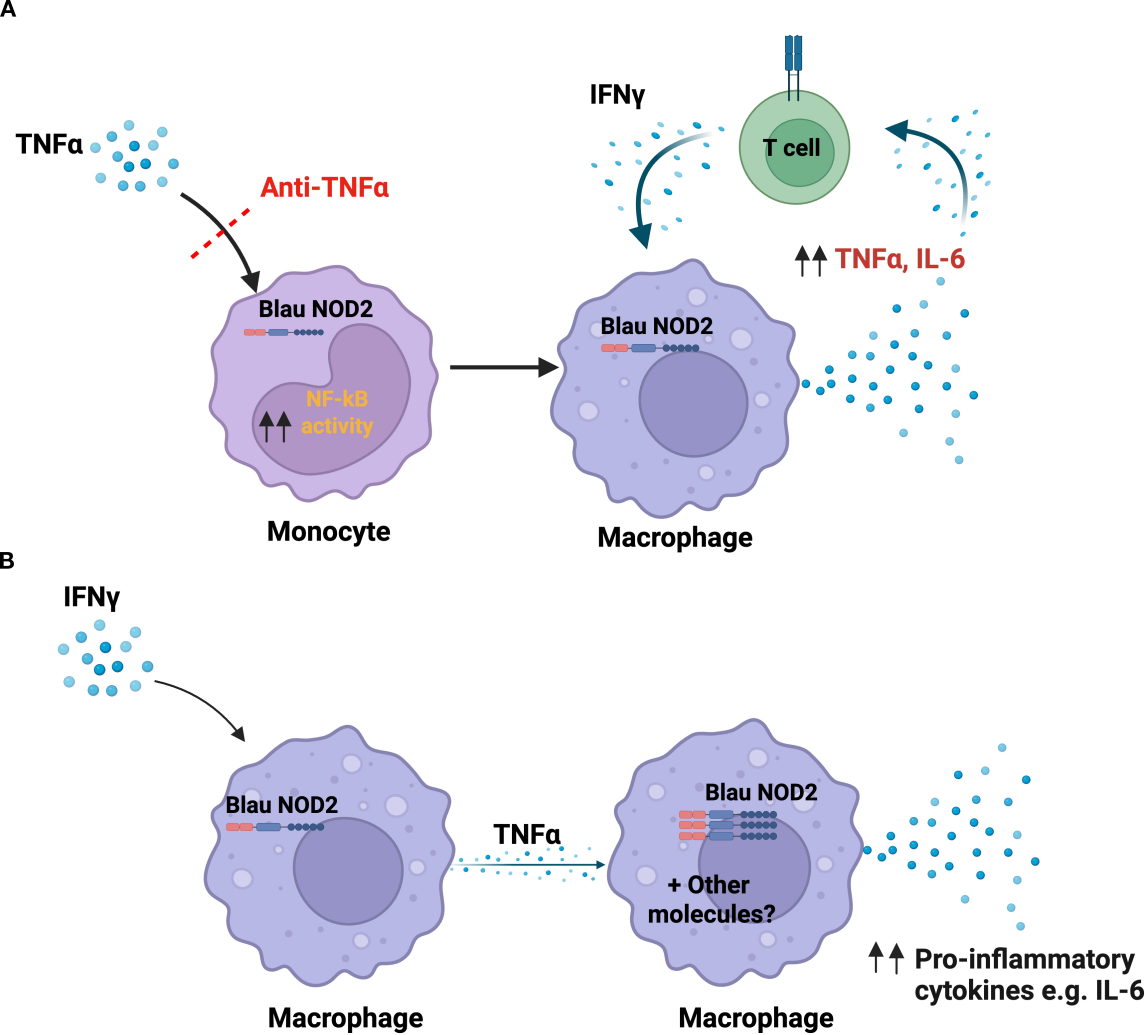
Role of the TNF-α and IFN- γ signaling in Blau syndrome. **(A)** Takada et.al., and Kitagawa et.al., found that Blau patient monocytes are abnormally sensitive to TNF-α signaling and its up-regulation of NF-κB activation. They therefore postulate that the macrophages derived from these monocytes are hyper-responsive to IFN-*γ* (plausibly derived from T/NK cells) and thus exhibit enhanced IFN-*γ*-induced production of IL-6 and TNF-α; this, in turn, causes T cell cytokine production (including IFN-*γ*) and the generation of an inflammatory feedback loop. Anti-TNF-α treatment of Blau patients can interrupt this pro-inflammatory state. **(B)** An alternative possible explanation for the observed TNF-α/IFN-*γ* responsivity of Blau monocytes described by the above authors that more clearly integrates NOD2 involvement is that IFN-*γ* and TNF-α act synergistically and stepwise to increase expression of mutant NOD2 and the latter is thus enabled to induce a hyper-inflammatory state (see text for further discussion). Image was generated using Biorender software.

Finally, it was observed that the macrophages of non-anti-TNF-α treated Blau patients displayed an increased M1 macrophage-associated gene signature upon IFN-γ treatment, in comparison to healthy controls. In further studies designed to overcome the environmental effects influencing the observations in primary human monocyte-derived macrophages the authors also performed similar RNA-seq based analyses with iPS cells derived from WT and Blau mutant macrophages. Here they observed that IFN-γ stimulated iPS cells derived mutant macrophages and macrophages from untreated Blau patients displayed a common set of 93 upregulated genes, associated with innate immune responses. However, the genes particularly enriched in the IFN-γ-stimulated Blau syndrome patient PBMC-derived macrophages included those involved in ‘Type I interferon signaling’ and ‘TNF-mediated signaling pathway’ (44) (**Figure 2A**).

The above studies by Kitagawa et.al., concerning the effects of IFN-γ and TNF-α on myeloid cells bearing a *CARD15* mutation are potentially important to our understanding of the mechanism of abnormal NOD2 function in Blau syndrome and, as such, need independent confirmation. Overall, they tend to strengthen the view that the Blau mutation in *CARD15* results in NOD2 that causes pro-inflammatory cell activation independently of its canonical ligand, MDP, but with the proviso that such activation is not initially mediated by NF-κB and the latter only occurs secondarily to the release of activating cytokines. Kitagawa et. al., explain their observations concerning TNF-α and IFN-γ effects on Blau macrophages by postulating that Blau monocytes are abnormally sensitive to TNF-α stimulation and respond to it by enhanced up-regulation of NF-κB; as a result, the macrophages derived from such activated monocytes are abnormally sensitive to IFN-γ and react to the latter with enhanced cytokine production. This, in turn, results in T cells production of IFN-γ that causes back stimulation of macrophages and the establishment of a forward feedback inflammatory loop (**Figure 2A**).

An alternative explanation of these findings that more clearly integrates the role of abnormal NOD2 into the IFN-γ effect and that also dispenses with the postulated IFN-γ-mediated up-regulation of NF-κB is that exposure of Blau macrophages to IFN-γ (or more precisely to IFN-γ and TNF-α) causes a major increase in the load of pathologic NOD2 in the cell as well as a possible increase in the latter’s distribution and exposure to cooperating factors. This, in turn, then leads to initiation of the actual (but as yet undefined) pathologic mechanism that ignites Blau syndrome inflammation. With this explanation, the finding that TNF-α signaling is necessary for Blau cells to manifest increased responsiveness to IFN-γ is assumed to be due to the fact that IFN-γ has only minor effects on NOD2 expression and function in the absence of TNF-α and the two cytokines have a synergistic effect. This alternative explanation is favored by the fact that it requires no elucidation of this as yet undefined GOF outcome of the Blau NOD2 function (**Figure 2B**). In addition, it allows the possibility that the GOF is secondary to a LOF defect involving a regulatory immune mechanism also undefined.

### IL-17 responses in Blau syndrome

It is now well established that NOD2 has general immunoregulatory properties in myeloid cells that cause down regulation of innate immune responses, particularly those in the GI tract where peptidoglycan (PGN) and its breakdown product, MDP, is abundant. This fact and its relation to Blau syndrome will be discussed in greater detail below. At this point we will turn to the fact that mice with NOD2 deletion exhibit heightened Th17-derived IL-17 production upon MDP stimulation (68); thus, it is now emerging that NOD2-mediated immunoregulatory function has a particular impact on Th17 T cell responses and this in itself may influence Blau syndrome pathogenesis.

NOD2 regulation of IL-17 responses was first noted in studies of uveitis in which it was found that mice with NOD2 deletion exhibited more severe eye inflammation upon ocular exposure to PGN and that the latter was associated with increased ocular IL-23 and IL-17 not IL-12 and IFN-γ responses (68). In the first of two later studies by Napier et al., addressing this issue it was observed that SKG mouse bearing a ZAP70 mutation causing increased susceptibility to arthritis upon challenge with a microbial beta-glucan, zymosan, exhibit more severe arthritis if they also have been subjected to NOD2 deletion (69). The heightened severity of arthritis in the NOD2^-/-^ SKG mice was concomitant with the accumulation of T cells, particularly CD4^+^ T cells, in the synovial fluid of inflamed joints and these synovial cells displayed an increased ability to produce IL-17. Furthermore, neutralization of IL-17 post arthritis induction resulted in a reduction in arthritis severity. Finally, CD4^+^T cells isolated from the zymosan immunized NOD2^-/-^ SKG mice had an inherent ability to induce more severe arthritis in nude recipient mice, suggesting a CD4^+^T cell-specific role in the IL-17-mediated exacerbation of the arthritis (69).

In the second report, Napier et. al., utilized a mouse model of experimental autoimmune uveitis (EAU) to understand the role of NOD2 in the development of intraocular inflammation. They observed that in the absence of NOD2, mice immunized with ocular self-antigen (IRBP) to induce uveitis displayed increased severity of uveitis associated with an increased accumulation of CD4^+^T cells in the eye. In studies to understand this effect they showed, particularly with studies of uveitis in Rag1^-/-^ mice reconstituted with T cells from NOD2^-/-^ and NOD2^+/+^ mice, that the increased susceptibility to EAU in the absence of NOD2 was due to the ability NOD2 to down-regulate CD4^+^T cell IL-17 production. In addition, they showed that this effect was independent of Th17 differentiation capacity, was down-stream of TCR stimulation and, surprisingly, did not occur as a result of RIPK2 signaling that mediates NOD2 responses in myeloid cells. Finally, they showed that this T cell intrinsic effect of NOD2 did not affect Th1/IFN-γ responses but did augment a host of Th17-related responses such as up-regulation of IL-23R, CCR7 and ROR-γT (70).

**Figure 3:**
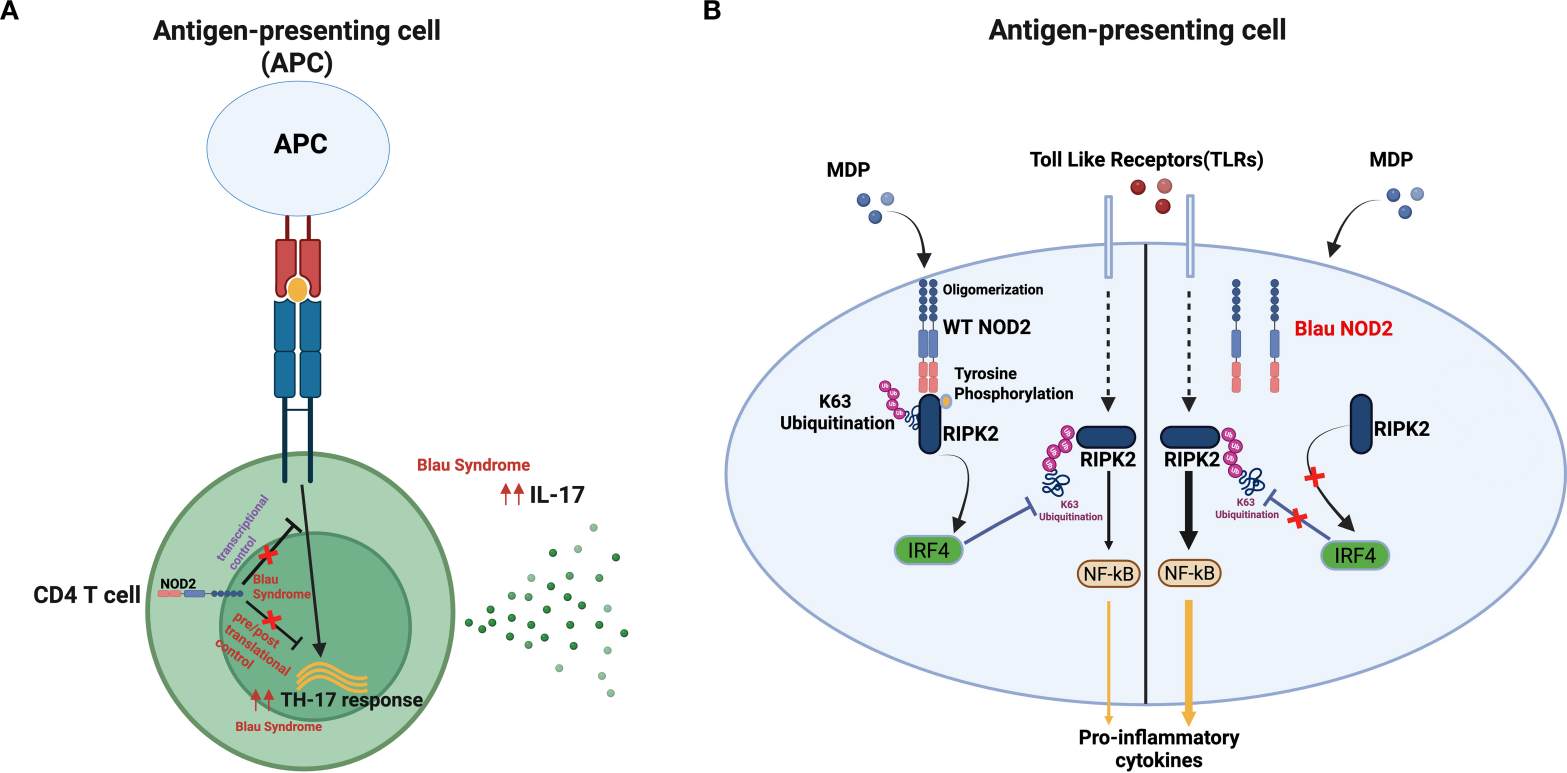
Immunoregulatory pathways in Blau syndrome. **(A)** Possible faulty regulation of the T cell IL-17 response by Blau NOD2: NOD2 has been shown to negatively regulate the Th17 response by CD4^+^ T cells initiated by the T cell receptor signalosome (70), through a yet undefined mechanism; such negative regulation might be defective in Blau syndrome and thus cause enhanced Th17 responses that contribute to Blau syndrome immunopathogenesis. **(B)** Possible faulty regulation of the myeloid TLR response by Blau NOD2: MDP stimulation of NOD2 and resultant RIPK2 signaling leads to enhanced IRF4 levels. IRF4, in turn, deubiquitinates RIPK2 (also acting downstream of TLRs) and thereby causes down-regulation of NF-κB activation (21, 71). Such negative regulation maybe defective in Blau syndrome, thus causing inflammation via enhanced TLR responses. Image was generated using Biorender software.

Overall, the two Napier et. al., studies summarized above provided strong evidence that NOD2 plays an immunoregulatory and negative role relating to IL-17 production by T cells that is likely independent of its regulatory role in myeloid cells discussed in greater detail below. However, the mechanism of this T cell regulatory effect, either with respect to NOD2 stimulation or to the latter’s function upon stimulation remains to be elucidated (**Figure 3A**). Finally, it should also be noted the negative effect of NOD2 in T cells shown in these studies of murine T cells needs to be considered in relation to a number of previous studies providing evidence that NOD2 signaling, contrary to the studies described above, has a positive effect on the Th17 response in a variety of immunological contexts (72–74). Most notably, van Beelen et.al., showed that stimulation of human dendritic cells with NOD2 ligand (MDP) enhances IL-17 production (but not IFN-γ production) by TLR-stimulated memory T cells and, in addition, enhances TLR-stimulated T cell differentiation into Th17 cells (74). Thus, the possible negative effect of NOD2 on IL-17 production in T cells may be counter-balanced by a positive NOD2 effect on dendritic cells.

Inasmuch as Blau syndrome is marked by reduced NOD2 responses, at least with respect to stimulation by its main ligand, MDP, it becomes likely that Blau syndrome, like mice with NOD2 deficiency, have increased IL-17 responses. Is this supported by already existent study? To answer this question, one must turn to the relatively limited body of studies that have probed cytokine responses in Blau syndrome. In one study in this category, granulomatous tissue from 6–7 Blau syndrome patients or Crohn’s disease patients with NOD2 risk polymorphisms were subjected to immune-histologic staining to estimate cellular cytokine production. Whereas patients in both groups exhibited strong IFN-γ staining of both monocyte/macrophage cells and T cells, the Blau syndrome patient cells exhibited greater IL-17 staining than the same cells from Crohn’s patients (75). This result, while consistent with the prediction that Blau syndrome leads to increased Th17 responses, is counter-intuitive since both Blau patients and Crohn’s patients have reduced MDP responsiveness. However, this paradox could be resolved by assuming that the NOD2 abnormality in Crohn’s disease does not affect its regulatory function in T cells.

Other studies of Blau patient cytokine responses providing data relative to their Th17 responses include studies of small numbers of patients showing that serum levels of IL-17 and IL-23 are particular increased in Blau patients (76); in addition, in one patient with uveitis, IL-17 was present in the ocular tissue (77) and in another patient circulating CD4^+^CCR4^+^ T cells (i.e., cells usually productive of IL-17) were increased (78). Finally, in one study, peripheral CD4+ T cells from two patients with Blau syndrome produced increased IL-17 upon TCR stimulation (70). Taken together, it is fair to say that there is positive, albeit limited, evidence that IL-17 responses are increased in Blau syndrome.

### Immunoregulatory abnormalities in Blau syndrome

It is well documented that NOD2 under normal circumstances exerts an immunoprotective host defense role in relation to bacterial as well as to viral and fungal pathogens (79). This is accomplished, at least in part, via its ability to respond to its ligand, MDP, with the RIPK2-mediated activation of NF-κB/MAPK signaling and subsequent production of a panoply of pro-inflammatory cytokines. However, there is also strong evidence (well summarized elsewhere) that this pro-inflammatory NOD2 role is accompanied by an anti-inflammatory or regulatory role (80). This became evident in studies of models of intestinal inflammation and patients with Crohn’s disease wherein NOD2 function is genetically impaired and, consequently one sees enhanced myeloid cell TLR-induced responses; in contrast, following administration of MDP to mice one sees amelioration of induced intestinal inflammation that depends on the presence of NOD2 (81). While the mechanism underlying such regulation is not yet fully determined there is evidence that it involves NOD2 activation of one or more de-ubiquitinating factors (DUBS) such as IRF-4 that impair NF-κB activation by preventing the necessary polyubiquitination of co-factors that enable NF-κB activation (71). Inasmuch as Blau syndrome, as argued above, is characterized by a gene mutation that causes loss of NOD2 function, at least in response to MDP, one has to consider the possibility that it also causes enhanced inflammation in Blau syndrome because of loss of regulatory function. What is the evidence that this is actually the case?

Studies conducted by Mao, Dhar and their collaborators have provided an organized set of data that bears on this question (21). In initial *in vitro* studies to understand the effects of Blau mutations on NOD2 signaling they evaluated the latter in HEK293 cells bearing constructs expressing WT NOD2 and NOD bearing Blau mutations. Here it was observed that the majority human Blau mutations studied caused reduced NOD2 oligomerization, poor interaction of NOD2 with RIPK2 and impaired RIPK2 mediated NF-κB activation. Then, in companion *in vivo* studies, they showed that whereas mice in which WT NOD2 was over-expressed were protected from induction of TNBS-colitis, mice in which Blau NOD2 was over expressed were not protected.

In further studies Mao, Dhar et al. employed Blau knock-in (KI) mice previously developed by Dugan et al. (45) to gather additional insight into Blau NOD2 signaling. As previously noted, these mice express a largely truncated form of mutated NOD2. However, it was shown that the truncated NOD2 interacts with full-length NOD2 and thus can affect the function of unmutated NOD2 present in heterozygous mice. In *in vitro* studies they found that dendritic cells from heterozygous Blau KI mice were similar to those from Blau syndrome patients in that they exhibit poor responsiveness to MDP stimulation leading to reduced downstream NF-κB and MAPK signaling compared to WT counterparts. However, signaling and cytokine responses of these dendritic cells to TLR ligands (LPS and Pam3Csk4) were heightened compared to WT dendritic cells. Then, in *in vivo* studies, they found that heterozygous Blau KI mice displayed more severe DSS induced colitis and anti-collagen antibody induced arthritis. Thus, both *in vitro* and *in vivo* Blau KI mice exhibited heightened pro-inflammatory responses reflecting a reduced NOD2 down-regulatory activity. Interestingly, this could be linked, as in the case of Crohn’s disease NOD2 loss-of-function mutations, to reduced expression of IRF4, the latter acting in the capacity of a deubiquitination factor that inhibits NF-κB activation both after *in vitro* cell stimulation and *in vivo* inflammation induction (**Figure 3B**).

The above studies by Mao, Dhar and their colleagues provide substantial evidence that the Blau mutation and its attendant loss of MDP-mediated signaling function is accompanied by loss of “cross-regulation” of TLR responses in myeloid cells and consequent enhancement of inflammatory responses. As mentioned, such loss is similar to that occurring in Crohn’s disease; however, whereas in the latter disease inflammation resulting from such loss is myeloid cell-extrinsic in the sense that it depends on the effects of gut microfloral stimulation, in Blau syndrome inflammation resulting from the loss is myeloid cell-intrinsic in the sense that it may accompany a pro-inflammatory effect of the mutated NOD2 not requiring ligand stimulation. This is to say that loss of myeloid cell cross-regulation may be a primary mechanistic cause of inflammation in Crohn’s disease whereas as in Blau syndrome it is could be a secondary and aggravating mechanistic cause of inflammation. Interestingly, recent studies of NOD2 regulation of IL-17 responses discussed above suggest that NOD2 also exerts regulatory function in T cells as well as in myeloid cells; whether similar mechanisms underlie both forms of regulation awaits further study but doesn’t seem likely given that myeloid regulation is MDP/RIPK2-dependent whereas T cell regulation is MDP/RIPK2-independent.

A final point concerning myeloid cell NOD2 cross-regulation presumably lost in Blau syndrome relates to the question of why such loss does not cause a Crohn’s disease-like intestinal inflammation in Blau patients. The answer to this question is unknown but one can speculate that Crohn’s disease occurrence may require the presence of a concomitant mucosal regulatory defect that licenses gut inflammation and that is not present in Blau syndrome patients.

## Discussion

The above analysis of the various aspects of the genetic abnormalities and immunologic defects associated with the granulomatous inflammation characterizing Blau syndrome clarifies a number of questions regarding the pathogenesis of this syndrome and points to the likely direction of further productive study. However, at the moment, we cannot say that this analysis applies to Yao syndrome, a “sister” *CARD15*/NOD2 genetic abnormality with a somewhat differing *CARD15* mutational locus and a distinctive clinical/pathological presentation; further work centering on immunologic abnormalities occurring in Yao syndrome will be necessary to enable such inclusion.

Perhaps the most important question concerning Blau syndrome pathogenesis addressed here is whether or not Blau syndrome is due to a NOD2 mutation that causes a gain-of-function NOD2 abnormality. Our analysis led to the conclusion that whereas structural studies provide fairly strong support for the view that NOD2 mutations can give rise to a NOD2 having the configuration of an activated molecule in the absence of MDP stimulation and/or a NOD2 less able to re-assume an inactive configuration following activation by MDP stimulation, the data supporting the idea that this results in spontaneous MDP-independent NF-κB activation is deeply flawed. Primarily, this negative assessment derives from the fact that whereas spontaneous NF-κB activation has been observed in some (but not all) studies of cells transfected with Blau NOD2-expressing plasmids and over-expressing Blau NOD2, one does not see evidence of spontaneous NF-κB activation in un-manipulated cells obtained directly from patients. This is not to say that Blau NOD2 is incapable of initiating another mode of gain-of-function not involving a direct activation of NF-κB. Indeed, there is some evidence derived from studies of TNF-α/IFN-γ stimulated myeloid cells that this does in fact occurs (44, 51), albeit by an unknown mechanism. Future investigation again addressing this question needs to provide evidence that a proposed gain-of-function effect was not, in reality, due to a more primary loss of regulatory function. As such, they need to focus on studies of cells freshly obtained from Blau syndrome patients and that are designed to discriminate between primary and secondary responses; in addition, such studies should be in search of novel interactions of Blau NOD2 with molecules having previously unrecognized pro-inflammatory potential.

A second question addressed in this review of Blau syndrome immunopathogenesis centers on the notion that Blau syndrome is simply another member of the family of autoinflammatory diseases due to excessive inflammasome activity. An essential feature of Blau syndrome immunologic dysfunction for this to be the case is that Blau NOD2 somehow co-opts the function of a *bona fide* inflammasome molecule to activate a caspase and thus create mature and secretable IL-1β and IL-18. The available evidence suggests that whereas normal NOD2 signaling may indeed lead to IL-1β secretion, possibly via activation of the NLRP1 inflammasome (53), Blau NOD2 signaling does not cause excessive IL-1β secretion (41, 42) and there is no indication that Blau NOD2 enters into an abnormal association with an inflammasome backbone molecule. Thus, there is little or no evidence that Blau syndrome is similar to diseases caused by excessive inflammasome function. Any further work along these lines would have to seek evidence of evanescent IL-1β responses that serve early and directing functions, rather than sustained and dominating functions.

Two relatively new and possibly interlocking ideas relating to mechanisms of Blau syndrome immune dysfunction are that Blau syndrome pathogenesis may reside in the disruption of NOD2 function as an immunoregulatory molecule in both myeloid cells and T cells. With respect to the former, its relation to myeloid cell immunoregulation, evidence was obtained that faulty MDP-mediated activation of NOD2 in Blau syndrome results in reduced production of a molecule ordinarily involved on TLR “cross-regulation” that results in increased pro-inflammatory TLR-induced cytokine production, as postulated to occur in Crohn’s disease (21). However, this explanation of Blau auto-inflammation, however elegant, falls short of explaining why sites of Blau syndrome and Crohn’s inflammations are quite distinct and, in particular, Blau syndrome is not a GI-centered disease. With respect to the latter, its relation to T cell immunoregulation, whereas the available data showing that NOD2 normally participates in TCR-initiated T cell IL-17 responses as a down-regulatory factor (70) is important and groundbreaking it does not address several key questions concerning T cell NOD2 responses. One is that it does not explain how TCR stimulation invokes a positive (albeit down-regulatory) NOD2 responses independently of MDP or other external NOD2 ligands of microbial origin; another is that it provides no clue as to how NOD2 down-regulates IL-17 production, other than the fact that such regulation does not involve RIPK2 signaling. The fact that NOD2 functions in T cells via its effect on IL-17 and the latter’s participation in eye and joint inflammation positions this NOD2 response as a likely factor in Blau immunopathology. However, the data documenting the presence of increased IL-17 responses in Blau syndrome patients supporting this possibility is sparse and requires further confirmation. In addition, given the fact that NOD2 signaling has been shown (in humans) to affect dendritic cell-induced Th17 differentiation it is important to investigate if the proposed Blau NOD2 T cell defect is not neutered by a Blau NOD2 dendritic cell defect. Finally, and parenthetically, if abnormal IL-17 responses are a major feature of Blau syndrome, the idea that the latter is not centered on NF-κB activation would be further substantiated.

## Conclusion

In conclusion, the possibility that loss of immunoregulatory function plays an over-arching role in the causation of Blau syndrome immunopathology is attractive because it spans findings relating to both myeloid cell and T cells in this autoinflammation. In addition, this possibility better fits with the fact that there is little evidence that Blau NOD2 mediates spontaneous pro-inflammatory cytokine production that is predicted by structural data showing that Blau NOD2 is in an activation configuration. This said, this concept has yet to explain why the Blau mutational defect does not result in the GI inflammation of Crohn’s disease, a disease also associated with a NOD2 genetic defect causing loss of NOD2 signaling. In addition, it has yet to account for the reported (but as yet unverified) increased responsiveness of Blau NOD2-bearing cells to TNF-α/IFN-γ stimulation. At this point, it is probably best to conclude that Blau syndrome is in fact due, at least in part, to a loss-of-function immunoregulatory abnormality, but whether this is coupled with a co-existing but uncharacterized gain-of-function abnormality still remains a possibility.
